# Bilateral femur osteomyelitis: A rare case with successful outcome

**DOI:** 10.12669/pjms.42.(ICON26).15702

**Published:** 2026-04

**Authors:** Shahzad Ahmed Mengal, Syed Ali Anwar Jilani, Nazir Alam

**Affiliations:** 1 Shahzad Ahmed Mengal, FCPS, FIOTA., Department of Orthopaedics, The Indus Hospital and Health Network, Karachi, Pakistan; 2 Syed Ali Anwar Jilani, FCPS., Department of Orthopaedics, The Indus Hospital and Health Network, Karachi, Pakistan; 3 Nazir Alam, FCPS., Department of Orthopaedics, The Indus Hospital and Health Network, Karachi, Pakistan

**Keywords:** Antibiotic-coated nail, Bone defect, Chronic osteomyelitis, Femur, Ilizarov technique, Limb salvage, SIGN nail

## Abstract

Chronic femoral osteomyelitis with segmental bone loss remains a complex reconstructive challenge, particularly in the setting of failed prior fixation and multidrug-resistant infection. Bilateral femoral involvement is extremely rare and lacks a standardized management algorithm. This report aims to describe a staged, limb-salvage strategy employing different reconstruction techniques for each femur based on defect size, biological potential, and infection status.

**Case Presentation:**

A 28 years old male presented with bilateral thigh pain and inability to ambulate three years after bilateral femoral shaft fractures complicated by recurrent surgical site infections and multiple revision surgeries. Clinical, laboratory, and radiological evaluation confirmed chronic bilateral femoral osteomyelitis with significant segmental bone defects, more severe on the right side.

**Management and Outcome:**

The left femur was managed with aggressive debridement and single-stage stabilization using an antibiotic-coated intramedullary SIGN nail, followed by culture-directed antimicrobial therapy for multidrug-resistant organisms, resulting in radiological union at six months. The right femur required staged reconstruction with debridement and Ilizarov-based distraction osteogenesis. Poor regenerate formation necessitated adjunctive teriparatide therapy, followed by definitive fixation using intramedullary nailing and augmentation with a non-vascularized fibular strut graft. At one-year follow-up, the patient was ambulating independently with bilateral radiological union and no evidence of recurrent infection.

**Conclusion:**

This case illustrates that individualized, staged reconstruction combining infection eradication, mechanical stability, and biological augmentation can achieve successful limb salvage in rare and complex cases of bilateral femoral chronic osteomyelitis.

## INTRODUCTION

Bilateral femoral shaft fractures are rare injuries, accounting for approximately 1-5% of all femoral shaft fractures, and are most commonly the result of high-energy mechanisms such as road traffic accidents.^1^ These injuries are often associated with extensive soft-tissue damage, prolonged periods of immobilization, and an increased risk of postoperative infection following surgical management.^2^ Chronic femoral osteomyelitis remains a particularly challenging condition to treat, with reported recurrence rates ranging from 10% to 30%, especially in patients with segmental bone loss, multiple prior surgical interventions, and failed internal fixation. Bilateral involvement is exceedingly uncommon, with only a limited number of cases reported in the literature.^2^,^3^

The management of chronic femoral osteomyelitis with associated bone defects necessitates a multidisciplinary and staged approach aimed at complete eradication of infection, restoration of skeletal stability, and biological reconstruction.^3^ Available treatment modalities include radical debridement with antibiotic spacers, antibiotic-coated intramedullary nails, external fixation with bone transport, the Masquelet induced membrane technique, vascularized and non-vascularized bone grafting, and hybrid strategies combining internal and external fixation. Despite the availability of these techniques, no universally accepted treatment algorithm exists, particularly for complex cases involving bilateral disease.^4^-^6^ This case report highlights a tailored limb-salvage strategy employing different reconstructive techniques for each femur, guided by defect size, local biological environment, and infection status.

## CASE PRESENTATION

A 28 years old male with no known comorbidities presented to the outpatient department in 2022 with complaints of bilateral thigh pain and inability to ambulate. He had sustained bilateral closed femoral shaft fractures in a road traffic accident in 2019. Initial management consisted of open reduction and internal fixation of both femurs using plate osteosynthesis. His postoperative course was complicated by bilateral surgical site infections, necessitating implant removal and prolonged antibiotic therapy. Over the subsequent years, the patient underwent multiple revision procedures involving reapplication of fixation devices followed by their removal due to recurrent infection.

**Fig.1:**
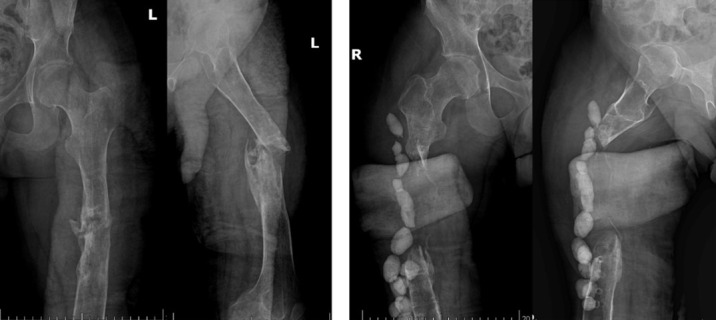
Preoperative radiographs of both femurs showing bilateral chronic non-union with segmental bone defects, more pronounced on the right side, and antibiotic beads in situ in the right femur: left femur AP and lateral views (left) and right femur AP and lateral views (right).

At presentation to our institution, the patient had bilateral long-leg splints *in situ*. Radiological evaluation demonstrated chronic non-union of both femurs with segmental bone defects, more pronounced on the right side, along with retained antibiotic beads in the right thigh (Fig.1). Laboratory investigations revealed an erythrocyte sedimentation rate (ESR) of 16 mm/h and a C-reactive protein (CRP) level of 3.8 mg/L. Based on clinical, radiological, and laboratory findings, a diagnosis of bilateral chronic femoral osteomyelitis with significant bone loss was established.

**Fig.2:**
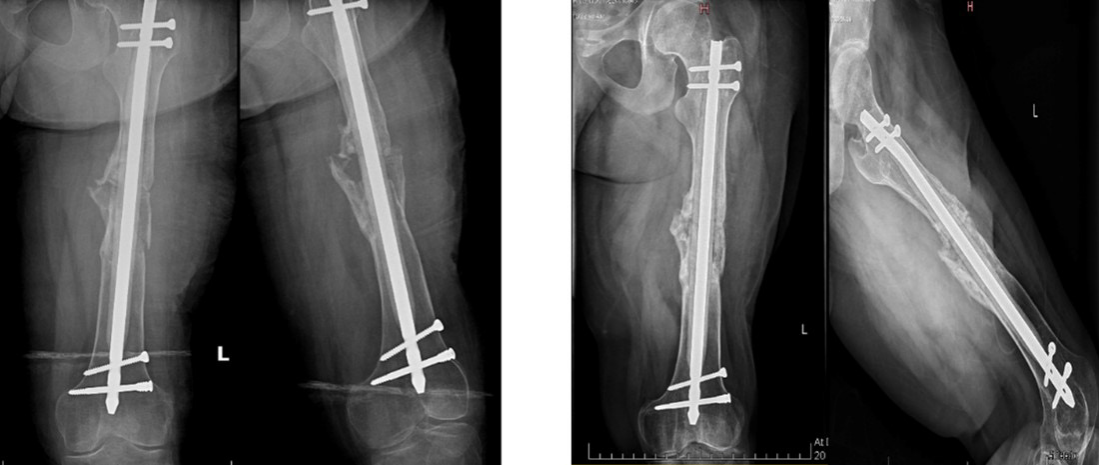
Immediate postoperative (left) and six-month follow-up radiographs (right) of the left femur demonstrating stabilization with an antibiotic-coated intramedullary SIGN nail and progressive radiological union.

Following multidisciplinary discussion, definitive surgical management of the left femur was undertaken first. A single-stage procedure was performed, which included aggressive debridement of fibrous tissue at the non-union site, freshening of bone margins, and procurement of tissue samples for histopathological examination and microbiological culture. A preoperative Gram stain was negative, following which stabilization was achieved using a solid intramedullary Surgical Implant Generation Network (SIGN) nail coated with heat-resistant antibiotic cement containing two gram of vancomycin. Culture and sensitivity results obtained after three days revealed methicillin-resistant *Staphylococcus aureus* (MRSA) and multidrug-resistant *Escherichia coli*. In consultation with the infectious disease team, the patient received intravenous tigecycline for two weeks, followed by oral trimethoprim–sulfamethoxazole (Septran DS) for a duration of three months. Histopathological analysis confirmed chronic osteomyelitis. Immediate postoperative and six-month follow-up radiographs are shown in Fig.2. Radiological evidence of union was noted at six months, with no clinical signs of infection, and the patient was allowed full weight-bearing on the left side.

**Fig.3:**
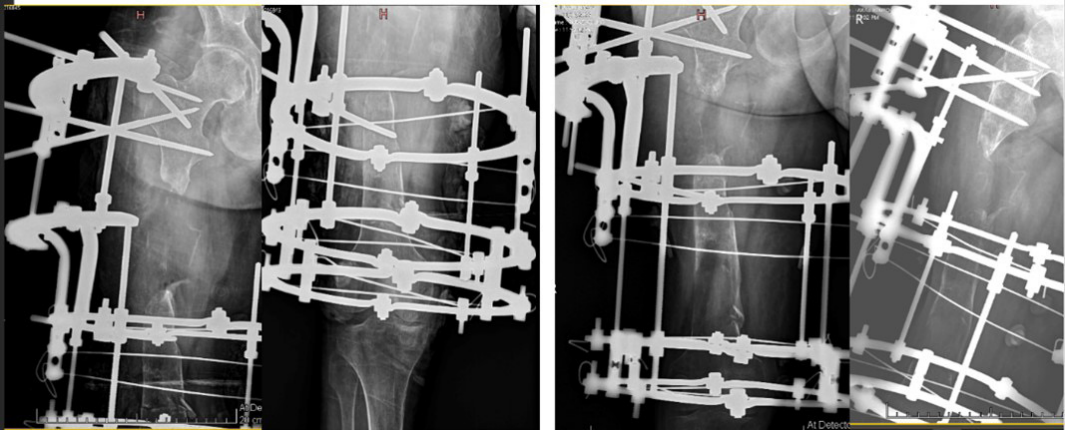
Right femur radiographs during Ilizarov-assisted distraction osteogenesis: immediate postoperative AP and lateral views (left) and 70 days follow-up AP and lateral views showing poor regenerate formation at the corticotomy site (right).

Subsequently, surgical management of the right femur was performed. The procedure involved thorough debridement with collection of tissue samples for culture and histopathology, freshening of bone ends, and application of an Ilizarov external fixator with a plan for bone defect reconstruction using the principle of distraction osteogenesis. Tissue culture grew multidrug-resistant *Escherichia coli*, for which intravenous meropenem (one gram three times daily) was administered for six weeks. Distraction was carried out at a rate of one mm per day, achieving a regenerate length of approximately 70 mm over 70 days. However, distraction was temporarily halted due to poor regenerate formation at the corticotomy site (Fig.3).

To enhance osteogenesis, the patient was initiated on anabolic therapy with subcutaneous teriparatide (parathyroid hormone analogue), along with calcium and vitamin D supplementation, for three months. Subsequent radiographs demonstrated improved regenerate quality with developing cortical formation. The Ilizarov frame was maintained for 12 months but was eventually removed due to grade four pin tract infection. The patient was again placed on suppressive antibiotic therapy, consisting of intravenous meropenem (one gram three times daily) for two weeks followed by oral trimethoprim–sulfamethoxazole for three months.

**Fig.4:**
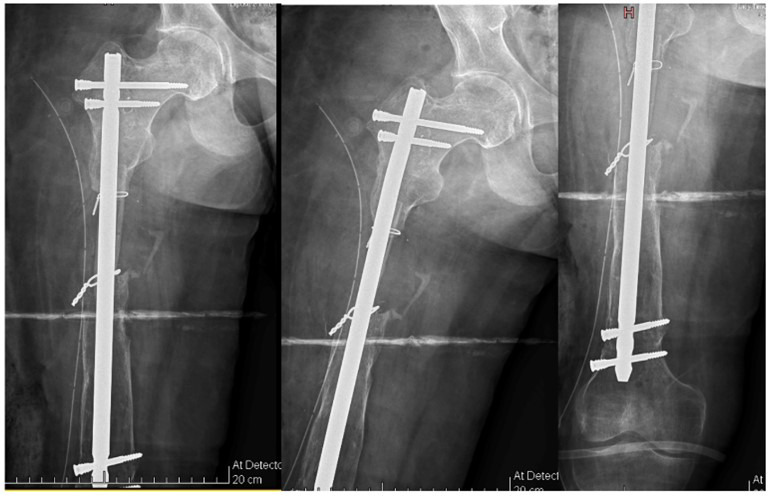
Postoperative radiograph of the right femur following definitive fixation with an intramedullary SIGN nail and reconstruction of the residual bone defect using a non-vascularized fibular strut graft.

After a six months infection-free interval confirmed clinically and radiologically, definitive fixation of the right femur was planned. Intramedullary stabilization was achieved using a SIGN nail, and the residual six cm bone defect was reconstructed using a non-vascularized fibular strut graft harvested from the ipsilateral limb during the same procedure (Fig.4).

**Fig. 5:**
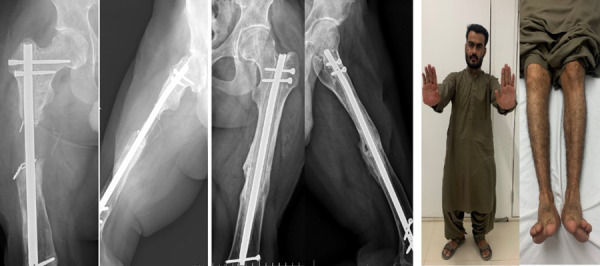
Follow-up images demonstrating successful outcome: right femur AP and lateral radiographs at one-year follow-up (left), left femur AP and lateral radiographs at two-and-a-half-year follow-up (middle), and corresponding clinical photograph showing functional ambulation (right), with maintained alignment and no evidence of recurrent infection.

The patient was followed for six months postoperatively, after which progressive radiological healing permitted advancement to full weight-bearing. At one-year follow-up, the patient was ambulating independently without limb length discrepancy, with confirmed bilateral femoral union on radiographs (Fig.5). Mild restriction of range of motion was noted in both lower limbs, more pronounced on the right side.

## DISCUSSION

Chronic femoral osteomyelitis associated with segmental bone loss remains one of the most demanding problems in orthopedic reconstruction, particularly in patients with a history of multiple failed surgeries and infection with multidrug-resistant organisms. The complexity increases further in bilateral disease, where treatment must balance infection eradication, mechanical stability, biological reconstruction, and functional recovery while minimizing morbidity. The fundamental objectives in such cases include complete control of infection, restoration of limb length and alignment, and achievement of durable bony union.

Several reconstructive options have been described for managing femoral bone defects following chronic osteomyelitis. The Masquelet induced membrane technique has shown favorable outcomes in defects up to five to six cm; however, it requires multiple staged procedures and may be less predictable in previously infected or extensively scarred tissues.^3^-^5^ Vascularized fibular grafts provide reliable biological reconstruction for large defects but demand microsurgical expertise and are associated with donor-site morbidity and prolonged operative time. Ilizarov-based distraction osteogenesis is widely regarded as the gold standard for large segmental defects, as it allows simultaneous bone regeneration and limb length restoration while maintaining stability in an infected environment. Nevertheless, this technique is associated with prolonged external fixation, pin-tract infections, joint stiffness, patient discomfort, and complications such as delayed or poor regenerate formation.^1^

In the present case, a side-specific and staged reconstructive strategy was adopted based on defect size, biological potential, and infection status. The left femur demonstrated a smaller bone defect and relatively favorable biological conditions, permitting single-stage aggressive debridement and stabilization using an antibiotic-coated intramedullary SIGN nail. This approach provided immediate mechanical stability and local antibiotic delivery, facilitating infection control and fracture union without recurrence. Increasing evidence supports the use of antibiotic-coated intramedullary nails as a viable alternative to traditional staged protocols in carefully selected cases of chronic osteomyelitis, particularly when stable fixation can be achieved following thorough debridement.^6^-^7^

In contrast, the right femur presented with a substantially larger defect measuring approximately 13cm and a compromised biological environment, necessitating reconstruction using Ilizarov-assisted distraction osteogenesis. Despite adequate distraction, regenerate formation was suboptimal, a well-recognized complication of bone transport, especially in the setting of chronic infection and repeated surgical insult. The use of teriparatide as an adjunctive biological agent in this case resulted in improved regenerate quality and cortical formation, consistent with emerging evidence supporting its role in enhancing osteogenesis in difficult nonunion and distraction scenarios.^8^,^9^ Subsequent conversion to intramedullary fixation with supplementation using a non-vascularized fibular strut graft provided additional mechanical stability and biological support, allowing definitive union while avoiding the morbidity associated with microsurgical reconstruction.

The distinguishing feature of this case is the successful application of two distinct reconstructive strategies in the same patient, tailored to the specific pathological and biological characteristics of each femur. Rather than adhering to a single reconstructive algorithm, treatment decisions were individualized, integrating infection control, mechanical principles, and biological augmentation. This approach resulted in bilateral union, functional ambulation, and absence of recurrent infection at one year follow-up.

This case highlights the importance of flexibility in surgical planning and supports a patient-specific, staged approach in the management of complex bilateral femoral osteomyelitis. Careful selection and combination of reconstructive techniques can achieve effective limb salvage and satisfactory functional outcomes, even in the most challenging clinical scenarios.

## CONCLUSION

Bilateral femoral chronic osteomyelitis with segmental bone loss is an exceedingly rare and challenging condition to manage. This case demonstrates that successful limb salvage can be achieved through a carefully planned, staged, and individualized treatment strategy emphasizing meticulous infection eradication, stable skeletal fixation, and appropriate biological augmentation. The selective application of single-stage antibiotic-coated intramedullary nailing for smaller defects and distraction osteogenesis with adjunctive biological support for larger defects resulted in bilateral union without recurrence of infection. Tailoring reconstructive techniques to defect size, local biology, and treatment response is essential for optimizing outcomes in complex cases of chronic osteomyelitis.

### Author contribution:

**SAM: S**tudy design, literature search, manuscript writing, data interpretation, and critical manuscript review.

**SAAJ:** Study concept and critical review.

**FZ:** Literature search and manuscript writing.

All authors have read the final version and are responsible and accountable for the accuracy and integrity of the work.
